# Exploring Relationships of Sleep Duration with Eating and Physical Activity Behaviors among Canadian University Students

**DOI:** 10.3390/clockssleep2020016

**Published:** 2020-05-26

**Authors:** Efrosini Papaconstantinou, Virginia Quick, Ellen Vogel, Sue Coffey, Andrea Miller, Hilde Zitzelsberger

**Affiliations:** 1 University of Ontario Institute of Technology, Faculty of Health Sciences, Oshawa, ON L1G 0C5, Canada; ellen.vogel@uoit.ca (E.V.); sue.coffey@uoit.ca (S.C.); andream@live.ca (A.M.); hilde.zitzelsberger@uoit.ca (H.Z.); 2 Rutgers University, School of Environmental and Biological Sciences, New Brunswick, NJ 08901, USA; vquick@njaes.rutgers.edu

**Keywords:** sleep, eating behaviors, physical activity, university students

## Abstract

Background: Students pursuing postsecondary education are a population described as vulnerable for sleep problems, poor dietary habits, weight gain, and reduced physical activity. The primary goal of this study was to examine relationships of sleep behaviors with eating and physical activity behaviors in a sample of undergraduate health sciences students. Methods: Using a cross-sectional design, undergraduate health sciences students in a small Canadian university were recruited to complete an on-line questionnaire about their sleep, eating, and physical activity behaviors using valid and reliable instruments. Key sociodemographic characteristics and self-reported height and weight data were also captured. Results: The participants (*n* = 245) were on average 23 years of age, female (86%), and the majority were full-time students (92%). The mean BMI was within a healthy range (mean 24.58 SD 5.55) with the majority reporting low physical activity levels (65%). Despite self-reports of very or fairly good (65%) sleep quality in the past month, the mean global sleep scores (scores > 5, mean 7.4, SD 3.3) indicated poor overall sleep quality. Poorer sleep quality was associated with higher BMIs (*r* = 0.265, *p* < 0.001). Conclusions: The findings highlight the need to expand the scope of on-campus wellness programs to promote healthy sleep habits in a vulnerable university population.

## 1. Introduction

An estimated 50 to 70 million adults globally suffer from sleep problems [1,2]. Consequences of sleepiness, disturbed sleep, and sleep disorders are associated with a multitude of poor health outcomes including weight gain and obesity-related comorbidities [3]. Insufficient sleep has been declared as a major public health problem by the Centers for Disease Control and Prevention (CDC) given its association with type 2 diabetes, heart disease, obesity, and depression [2]. Students pursuing postsecondary education are a population described as vulnerable for sleep problems [4], poor dietary habits and weight gain [5], and reduced physical activity [6,7].

For young adults (i.e., 18–25 years), the National Sleep Foundation (a U.S. non-profit organization) guidelines recommend seven to nine hours of sleep per night, stating that less than six hours of sleep per night can compromise health and well-being [8]. Despite adequate sleep being critically important for health, and in light of the fact that adults spend more than 30% of their lives sleeping, the majority of university students do not achieve the recommended amount of sleep each night, and the overall hours of sleep in this population has continued to decrease over the past decades. From 1969 to 2001, the median hours of sleep for postsecondary students decreased from eight hours to less than seven hours per night with an increased prevalence of self-reported dissatisfaction with sleep quality [9]. Irregular sleep habits and other sleep problems are prominent in university students. For example, over 60% of U.S. university students were categorized as poor-quality sleepers and had shortened sleep duration on weekdays, but delayed mean bedtimes (1:44 a.m.) and wakeup times (10:08 a.m.) on the weekends. In this population, poor quality sleepers reported shortened sleep duration on weekdays, with delayed mean bedtimes (1:44 a.m.) and wakeup times (10:08 a.m.) on the weekends [4].

Both biological and lifestyle factors contribute to erratic sleep schedules and sleep deprivation in university students. Physiologically, both adolescents and young adults tend to have a biologically driven delayed sleep phase, also referred to as a delayed circadian preference, contributing to their staying up later at night and to their being described as “night owls” [10,11]. This change occurs in association with puberty [10]; sex differences in circadian rhythm have been noted to change with age [12,13]. Changes in circadian rhythm that involve shifts from morningness (early bedtime and wake time preferences) to eveningness (late bedtime and wake time preferences) are common among university students [14]. Furthermore, students tend to experience less exposure to natural light [15]. The erratic sleep-wake schedules contribute to sleep deprivation, as many university students go to sleep late and wake up early to attend classes, thus starting their days before adequate sleep is achieved. In a sample of 236 college students who completed interview surveys, only 24% reported getting adequate nocturnal sleep with 79% reporting bedtimes after midnight [16]. Students attempt to compensate for suboptimal sleep by increasing sleep on the weekends. This practice, in some cases, can provide temporary relief, but in actuality overcompensation worsens the problem [17]. On weekends, compensatory oversleeping behavior leads to further disruptions in the sleep-wake cycles, exacerbates the normal adolescent circadian phase delay, and compromises weekday alertness [18]. Apart from biologically driven sleep delays, other factors such as smartphone devices and access to new media, lack of parental supervision, changed living environment on campus, overconsumption of caffeine, including caffeinated energy drinks, and academic stress can also contribute to irregular sleep habits in university students [4,9,19].

Irregular sleep-wake patterns and poor sleep quality are associated with increased daytime sleepiness and also with changes in endocrinology and metabolism [20]. In a systematic review and meta-analysis, poor sleep duration resulted in increased energy intake, leading to a net positive energy balance of 385 kcal per day, which, over time, could contribute to weight gain [21]. Previous work has suggested sleep deprivation can cause dysregulation of the metabolic hormones leptin and ghrelin which are key regulators of hunger and satiety [22,23], as well as executive function involved with the internal regulation of food [24,25], thereby leading to an increase in energy intake. Evidence suggests a more plausible explanation for the observed increase in energy intake after poor sleep duration is hedonically driven [26], that is, short sleep heightens the motivation to seek food as a reward. For instance, a study among U.S. university students reported that short sleep duration was associated with poor internal regulation of food intake and binge eating [25] which could explain the hedonic drive for food with a lack of sleep. Furthermore, short sleep duration was identified as a predictor for obesity [27,28,29]. A meta-analysis of studies on the association between short sleep duration and obesity found an increase in the odds of obesity for short duration sleepers (OR = 1.55, 95% CI = 1.43 to 1.68, *p* < 0.001) [30].

Findings from examining the association between sleep duration and physical activity have been mixed. Among university students, some research has found associations of sleep duration with physical activity [31,32] whereas other studies have not found any associations [25]. The mixed findings can be due to differences in populations under study and the types of physical activity measures used. However, several studies have supported the positive effects of physical activity on sleep. A systematic review found positive effects of both anaerobic and resistance exercise of various durations and moderate-to-high intensities on sleep in middle-aged and older adults [33]. Thus, engaging in physical activity can help university students sleep better at night while also contributing to healthy body weights.

Given that sleep duration and quality have many health benefits, the purpose of our cross-sectional study was to explore relationships of Canadian university students that meet and do not meet sleep duration recommendations set by the National Sleep Foundation (i.e., seven to nine hours/night), with respect to their eating and physical activity behaviors.

## 2. Materials and Methods

This cross-sectional study investigated sleep, eating, and physical activity behaviors among health sciences undergraduate students at a small university, located in Oshawa, Ontario, Canada. The Faculty of Health Sciences provides diverse undergraduate programs such as Nursing, Allied Health Sciences, Kinesiology, Health Sciences, and Medical Laboratory Science. After obtaining research ethics approval from the university, students were invited via e-mail through a central research listserv to participate in an online questionnaire via LimeSurvey^TM^ during the winter of 2018. The email invited students to participate in a short survey with questions about their sleep, eating, and physical activity behaviors. As an incentive, students could enter into a draw to win one of five gift cards valued at CAD $50.

### 2.1. Measures

The online survey included valid and reliable measures that assessed sleep, eating, and physical activity, and measures of sociodemographic and health characteristics as described further below.

#### 2.1.1. Sleep Behaviors

The 19-item, Pittsburgh Sleep Quality Index (PSQI) was used to assess patterns of sleep dysfunction over the past month and provide a global sleep quality index score that categorized an individual into a poor or good quality sleeper (scores > 5 = poor quality sleeper) [34]. A total of seven subscales comprise the PSQI as follows: (1) subjective sleep quality (i.e., perceived sleep quality); (2) sleep latency (i.e., usual time it takes to fall asleep at night); (3) sleep duration (i.e., average hours a person sleeps per night); (4) habitual sleep efficiency (i.e., actual hours of sleep compared to hours spent in bed); (5) Sleep disturbance (i.e., factors related to waking up during the night or early morning); (6) sleep medication use (i.e., the use of “over the counter” or prescribed medicines to help sleep); and (7) daytime dysfunction (i.e., difficulty in staying awake during the day). Composite scores of all seven subscales were summed to generate a sleep quality global index score. Among college students, the PSQI has shown good reliability and validity [4,25].

The Epworth Sleepiness Scale (ESS) was used to assess daytime sleepiness [35]. The 8-item, ESS provided a measure of excessive daytime sleepiness associated with accumulated sleep deprivation. Questions on the ESS asked participants to rate, on a 4-point scale (scored 0 to 3), their usual chances of falling asleep in different situations. Responses were summed for a total possible score range of 0 to 24; higher scores indicate greater daytime sleepiness. Scores over 10 are indicative of significant levels of daytime sleepiness.

#### 2.1.2. Eating Behaviors

The eating behaviors assessed were cognitive restraint, uncontrolled eating, and emotional eating using the Three Factor Eating Questionnaire (TFEQ-18) [36]. The 18-item TFEQ [37] is comprised of three scales that assess cognitive and behavioral components of eating. The cognitive restraint scale (six items) measures intentions to limit food intake for weight management purposes. The uncontrolled eating scale (nine items) evaluates uncontrolled eating behaviors. The emotional eating scale (three items) evaluates the influence of emotions on the urge to eat. Responses range on a 4-point scale from “definitely false” to “definitely true.” Items for each scale are averaged for a mean score with higher scores indicating greater restraint, uncontrolled eating, and emotional eating.

Daily fruit and vegetable intake and alcohol and caffeine intake per week were measured in the following ways. The valid and reliable 19-item National Cancer Institute Daily Fruit and Vegetable Screener assessed daily fruit and vegetable servings over the past month [38]. An algorithm was used to estimate the usual intake of fruits and vegetables eaten daily (in cup equivalents). Caffeine intake (servings/week) was derived from five questions, created de novo, asking participants to report the total servings per week of coffee, espresso, tea, soft drinks, and energy drinks. Responses were summed to determine a usual intake per week of caffeinated beverages. Alcohol intake was derived from four questions created de novo that asked participants to report total servings per week of beer, wine, liquor, and mixed drinks. Responses were summed to determine a usual intake per week of alcoholic beverages.

#### 2.1.3. Physical Activity Behaviors

Physical activity was assessed using a streamlined, enhanced self-reported physical activity measure adapted from the International Physical Activity Questionnaire (IPAQ) [39,40]. This index is based on the days per week (0 to 7 days) the following activities are completed: vigorous activities (e.g., heavy lifting, digging, aerobics); moderate activities (e.g., carrying light loads, bicycling at a regular pace); walking for at least 10 min at a time; and strength training (e.g., lifting weights, sit-ups). An index score was calculated with vigorous activity weighted higher than other types of activities and moderate activities weighted higher than walking and strength training to reflect relative differences in calorie expenditure of these activities. The physical activity index scores ranged from 0 to 49 with scores of <20 indicating sedentary levels, ≥20 to <30 indicating moderate levels, and ≥40 indicating high activity levels. This tool has been validated within the young adult population [25].

#### 2.1.4. Sociodemographic and Health Characteristics

Participants answered a range of questions that focused on demographics such as sex, race/ethnicity, age, residency, and year in academic program. Self-reported height and weight were collected to determine weight status using the body mass index (BMI) (height (m2)/weight (kg)) [41]. Two additional questions were created de novo and inquired whether or not participants regularly used over-the-counter (OTC) stimulants or sleep aids, as well as the reasons for taking them.

### 2.2. Data Analysis

All data were analyzed in the Statistical Package for Social Sciences 26 (IBM Corporation, Chicago, IL, USA). Participants that completed all items on the PSQI measure and self-reported height/weight data were included in this exploratory analysis (*n* = 245). To assess the reliability of study measures, internal consistency using Cronbach’s alpha were conducted. Descriptive statistics of all measures were performed for all participants. Given that prior work reported nonlinear associations of eating and physical activity behaviors with reported sleep duration, self-reported hours per night of sleep was categorized into those meeting and not meeting sleep recommendations (7 to 9 h per night) for adults.

Independent *t*-tests for continuous variables and Chi-square tests for categorical variables were performed to determine significant (*p* < 0.05) sociodemographic and health characteristic differences between those participants that met and did not meet sleep recommendations. Further analyses examining various sleep behavior (ESS, Global PSQI score, and use of stimulants) differences by those that met and had not met sleep recommendations were also conducted using independent t-tests and Chi-square tests. Lastly, adjusted binary logistic regression analyses controlling for BMI were performed to assess whether eating and physical activity behaviors were significantly associated with those that met sleep recommendations. Beta standard errors, odds ratios (OR), and 95% confidence intervals (CI) were computed for each independent variable in regression analyses examining associations with those that met sleep recommendations.

## 3. Results

Of the 245 participants that completed the survey, most were female (86.1%) and full-time students (92.2%) (Table 1). The sample was fairly diverse in terms of race (54.7% White, 28.6% Asian, 5.7% Black, and 8.6% mixed/other), year in university, and home program within the Faculty of Health Sciences (26.9% Collaborative Nursing Science (BScN), 22% Kinesiology, 21.6% Bachelor of Health Sciences, 11.8% Medical Laboratory Science, 9.8% RPN to BScN nursing program, and 6.5% Allied Health Science BAHSc). Additionally, almost two-thirds (64.1%) of participants were employed with one-third of those employed (33.5%) working 13 or more hours per week. Internal consistency scores for all measures were good (α ≥ 0.82), except for the PSQI (α = 0.68) that was slightly lower but still acceptable.

On average, participants were slightly under the recommended hours of sleep per night (6.80 ± 1.51 h/night) (Table 1). About half of the participants (*n* = 115, 46.9%) did not meet the sleep recommendations of seven to nine hours per night. Those that did not meet sleep recommendations were significantly (*p* < 0.01) more likely to be employed, work more hours per week, and have a higher BMI (25.60 ± 6.41 SD vs. 23.68 ± 4.49 SD) and age (24.28 ± 8.35 SD years vs. 21.67 ± 5.06 SD years) as compared with participants (*n* = 130) that met the sleep recommendations.

In terms of sleep behaviors, sleep quality was poor among all participants; the average sleep quality index score (7.41 ± 3.33 SD) was above the cut-off score of five, indicating poor sleep quality (Table 2). Over one-third of participants were categorized as being at high-risk for daytime sleepiness and self-reported their sleep quality as being very or fairly bad over the last month. Habitual sleep efficiency (i.e., number of hours slept/number of hours spent in bed × 100) was highest among most participants (82%), indicating good sleep efficiency at night; however, those not meeting sleep recommendations had significantly (*p* < 0.001) lower habitual sleep efficiency as compared with those meeting sleep recommendations. As expected, those not meeting sleep recommendations had significantly poorer sleep quality (both objective and subjective measures) and daytime sleepiness as compared with those meeting sleep recommendations. Of the 18 participants that reported regularly using OTC stimulants or sleep aids, most reported taking them on a regular basis for the following reasons: facilitate sleep (89%), increasing wakefulness or alertness (22%), weight loss or muscle gain/enhance performance (22%), and other health-related reasons (17%). Although not statistically significant, participants (*n* = 18) that regularly used OTC stimulants or sleep aids were twice as likely to not meet sleep recommendations.

Most participants, were not meeting the seven or more, daily recommended fruit and vegetables servings (2.44 ± 1.82 SD per day) (Table 3) (recommended servings based on the most current (2007) Canada’s Food Guide at the time of the study) (Health Canada, Eating Well with Canada’s Food Guide, 2007). On average, participants consumed approximately three servings of caffeinated beverages per week and approximately 1.5 servings of alcohol per week. Physical activity was low among all participants suggesting a sedentary lifestyle. Adjusted binary logistic regression controlling for BMI found no statistically significant differences in any of the eating and physical activity behaviors between participants that met and did not meet sleep recommendations. However, a non-significant trend revealed that participants not meeting sleep recommendations were more likely to report greater emotional eating and uncontrolled eating behaviors as compared with their counterparts meeting the sleep recommendations. BMI was statistically significant in all of the adjusted binary logistic regression models.

## 4. Discussion

This study explored the relationships of Canadian university students that met and did not meet sleep duration recommendations set by the National Sleep Foundation (seven to nine hours of sleep per night) with eating and physical activity behaviors. While the National Sleep Foundation recommends seven to nine hours of sleep for young adults, our study found that only just over half (*n* = 130, 53%) of our sample were meeting these recommendations. Reports from the CDC noted that only ~32% of those aged 18–24 years achieve the recommended amount of sleep [42]. In the USA, there is a high prevalence of low sleep duration among college students. In a sample of over 7600 U.S. college students across six universities, 36% of study participants reported less than seven hours of sleep per night [43]. Similarly, Quick et al. (2016) found that more than one quarter (28%) of participants (*n* = 1252) had inadequate sleep (<seven h/night) [25].

Upon further examination of sleep duration with BMI, our study found that those not meeting the sleep recommendations had a significantly higher BMI as compared with those meeting the recommendations (seven to nine hours/night). A recent meta-analysis found long sleep duration (>nine hours/night) to be associated with an increased risk of obesity in adults [44,45]. Unfortunately, our study was limited in being able to examine these similar relationships of short and long sleep duration with BMI due to the small sample size; however, future studies should consider examining these comparisons among postsecondary students.

Not surprisingly, those that met sleep recommendations reported better sleep quality and less daytime sleepiness than those not meeting the recommendations. The mean global PSQI score was 7.41 (SD 3.33) which is well above the cut-off score of five, indicating that students had poor overall sleep quality. This is consistent with studies across Europe and North America which have continued to find poor sleep quality at alarming levels among the postsecondary student population [4,46,47,48]. A consequence of poor quality sleep and sleep insufficiency is daytime sleepiness. Our study found that a significant proportion of students not meeting the sleep recommendations were more likely to report increased daytime sleepiness. Daytime sleepiness is the inability or difficulty in maintaining alertness during the major wake period of the day, resulting in unintended lapses into drowsiness or sleep [49]. This is concerning because daytime sleepiness negatively impacts learning, memory, and performance [19], and has been associated with an increased rate of motor vehicle collisions within the college student population [50]. Previous research has found significant sex differences in sleep habits and duration during weekdays and weekends in young adult populations [51,52]. In college students, females have a tendency to go to bed earlier and rise earlier, sleep longer, and report poorer sleep quality than male students [51,53]. Given our small sample size, we were unable to stratify our analyses by sex; however, future studies should consider sex and age differences in sleep outcomes.

Although our study found no differences in eating and physical activity behaviors between those that met and did not meet sleep recommendations, there could be a number of possibilities for these findings. Prior work in a cross-sectional analysis study has found sleep duration to have a nonlinear association with fruit and vegetable consumption, whereby short and long duration sleepers consumed less fruit and vegetables as compared with those sleeping seven to eight hours per night in U.K. adults [54]. A follow-up prospective study, in this same population, assessed the nonlinear associations of fruit and vegetable intake (measured by four-day food diaries) with sleep duration and modelled the association using restricted cubic splines and had similar findings [55]. This would suggest an inverse U-shaped association between sleep duration and fruit and vegetable consumption, a nonlinear association. Prior studies have found similar U-shaped associations of alcohol intake and physical inactivity with short and long duration sleepers [56,57], which could explain the non-significant findings in our study. Thus, future work should examine these nonlinear relationships between sleep and diet among college students. It is also possible that self-reporting of eating and physical activity behaviors could have biased our results given that individuals tend to underreport their eating and physical activity behaviors.

Furthermore, our study did not find any significant associations between the cognitive and behavioral components of eating (e.g., cognitive restraint, uncontrolled eating, and emotional eating) and sleep recommendations after controlling for BMI. Studies have found short sleep duration can be linked to weight gain and the development of obesity through its association with unhealthy eating patterns including increased intakes of energy and fat, increased ratio of fat to protein [58,59], and excessive calorie consumption from snacks [60]. Insufficient sleep can negatively affect regulation of food intake, making unhealthy food choices more appealing and rewarding [61]. In a study conducted with female university students (*n* = 410) in Iran, Haghighatdoost et al. (2012) found that participants with a sleep duration of less than six hours a day had a higher intake of energy, consumed significantly more carbohydrates, and significantly less protein than those with a sleep duration of more than eight hours day (2406 ± 825 versus 2092 ± 700 kcal) [62]. One possibility for our non-significant findings of the relationship between eating behaviors and sleep can be attributed to food insecurity. Poor sleep health is significantly associated with being food insecure [63]. Food insecurity has been defined as inadequate access to sufficient nutritious food [64] and is a public health issue [65]. A recent growing concern is the high reported prevalence of food insecurity among postsecondary students [66]. Some of the contributing factors to food insecurity within the postsecondary student population could be due to low income, rising tuition, housing, and food costs [67], as well as insufficient time (e.g., to prepare and consume a sufficient quality and quantity of food) and limited access to culturally appropriate foods [68,69]. Students use a number of coping strategies for food insecurity such as changing eating behaviors, reducing the size of the meals, and skipping meals [70]. Although our study did not assess food insecurity, future studies should consider including this emerging area as food insecurity can be a mediating factor that can influence the relationship between sleep and eating behaviors.

Our results should be interpreted in light of the following limitations. The small sample size and exploratory nature of this study precludes any definitive conclusions about the interconnections of sleep behaviors with eating and physical activity behaviors. Due to the exploratory nature of this study, it only identifies associations and not causal inferences. More longitudinal studies are needed in this emerging area. The low response rate within this study could be attributed to a labor disruption occurring at the time of the launch of the study. However, there were no differences noted between responders and non-responders. Finally, there are limitations of self-reported measures, as well as the possibility of residual confounding by unmeasured variables.

## 5. Conclusions

In conclusion, findings from this study suggest that a majority of postsecondary health sciences students attending a small Canadian university are not meeting sleep recommendations and have poor eating and physical activity behaviors. The transition from secondary school to postsecondary education can be characterized as a challenging time period and can be accompanied by unhealthy behavioral changes [71,72]. Campus health interventions should be a high priority in promoting healthy self-care behaviors in relation to sleep, eating, and physical activity behaviors.

## Figures and Tables

**Table 1:** Sociodemographic and health characteristics among Canadian college students (*N* = 245).

	All Participants		Sleep Recommendation Groups				
			Sleep Recs NOT Met (*n* = 115)		Sleep Recs Met (7 to 9 hr/night) (*n* = 130)		
Characteristic	N	%	N	%	N	%	*p*-value *
Sex ^‡^							0.338
Female	211	86.1	102	88.7	109	84.5	
Male	33	13.5	13	11.3	20	15.5	
Race/ethnicity ^a^							0.153
White	134	54.7	62	53.9	72	58.1	
Asian	70	28.6	40	34.8	30	24.2	
Black	14	5.7	7	6.1	7	5.6	
Mixed	11	4.5	2	1.7	9	7.3	
Other	10	4.1	4	3.5	6	4.8	
Enrollment status ^b^							0.127
Part-time	17	6.9	11	9.6	6	4.7	
Full-time	226	92.2	103	90.4	123	93.3	
College year ^b^							0.729
First year	48	19.6	19	16.5	29	22.7	
Second year	68	27.8	32	27.8	36	28.1	
Third year	64	26.1	34	29.6	30	23.4	
Fourth year	57	23.3	27	23.5	30	23.4	
Part-time or off track	6	2.4	3	2.6	3	2.3	
Employed (% yes) ^c^	157	64.1	85	75.2	72	57.6	0.004
Employment (hours/week) ^d^							0.002
Do not work	80	32.7	27	23.9	53	42.4	
≤12 h	76	31.0	36	31.9	40	32.0	
13 to <24 h	45	18.4	24	21.2	21	16.8	
≥24 h	37	15.1	26	23.0	11	8.8	
Marital status ^e^							0.227
Single	207	84.5	92	81.4	115	89.1	
Married or common law	32	13.1	19	16.8	13	10.1	
Divorced, separated or widowed	3	1.2	2	1.8	1	0.8	
Resident status ^e^							0.258
On-campus or off-campus (independent living)	97	39.6	50	43.9	47	36.7	
Off-campus (at home with family)	145	59.2	64	56.1	81	63.3	
	Mean	SD	Mean	SD	Mean	SD	*p*-value ^†^
Mean age (years) ^f^	22.88	6.90	24.28	8.35	21.67	5.06	0.005
Body mass index (kg/m^2^)	24.58	1.83	25.60	6.41	23.68	4.49	0.008
Self-reported sleep (hours/night)	6.80	1.51	5.83	1.58	7.64	0.76	<0.001

Note: Recs = recommendations, * Chi-square test for categorical values, ^†^ Independent *t*-tests for continuous values, **^‡^** 1 participant preferred not to disclose their gender, ^a^ N = 239 with 6 missing reported data, ^b^ N = 243 with 2 missing reported data, ^c^ N = 238 with 7 missing reported data, ^d^ N = 237 with 8 missing reported data, ^e^ N = 242 with 3 missing reported data, ^f^ N = 241 with 4 missing reported data.

**Table 2:** Sleep behaviors by Canadian college student groups that met and did not meet sleep recommendations (*N* = 245). Recs = recommendations.

	All Participants		Sleep Recommendation Groups						
			Sleep Recs NOT Met (*n* = 115)		Sleep Recs Met (*n* = 130)				
Characteristics	Mean	SD	Mean	SE	Mean	SE	t *	*p-*value	
Global PSQI score ^A^	7.41	3.33	9.30	0.31	5.74	0.20	9.72	<0.001	
Epworth Sleepiness Total ^B^ (*n* = 244)	8.80	4.79	10.26	0.45	7.49	0.38	4.68	<0.001	
	N	%	N	%	N	%	ꭕ^2 †^	*p-*value	
Daytime Sleepiness Risk (*n* = 244)							11.20	0.001	
Low risk	158	64.8	62	53.9	96	74.4			
High risk	86	35.2	53	46.1	33	25.6			
Subjective sleep quality (*n* = 244)							34.87	<0.001	
Very or fairly good	159	64.90	53	46.1	106	82.2			
Very or fairly bad	85	35.10	62	53.9	23	17.8			
Habitual sleep efficiency (*n* = 244)							25.21	<0.001	
≥75%	201	82.04	79	69.3	122	93.8			
<75%	43	17.55	35	30.7	8	6.2			
Regular use of over the counter (OTC) stimulants or sleep aids (% yes)	18	7.35	12	10.4	6	4.6	2.61	0.201	

* Independent *t*-tests examined sleep behavior characteristic differences between participants that met (7 to 9 h of sleep/night) and did not meet sleep recommendations for adults; ^†^ Chi-square analysis examined sleep behavior characteristic differences between sleep duration categories; ^A^ measured by the Pittsburgh Sleep Quality Index (PSQI), higher scores indicate poorer sleep quality (Cronbach α = 0.68); ^B^ higher scores indicate higher levels of daytime sleepiness (Cronbach α = 0.82), high risk for daytime sleepiness has a cut-off score of >10 on the Epworth Sleepiness scale; ^C^ habitual sleep efficiency (%) = (number of hours slept/number of hours spent in bed) × 100, higher scores indicate better sleep efficiency at night.

**Table 3:** Adjusted binary logistic regression of weight-related behaviors by Canadian college student groups that met and did not meet sleep recommendations. *N* = 245.

Scale (Possible Score Range) Subscale	Cron-Bach’s α	All Participants		Sleep Recommendation Groups					*p-*Value
				Sleep Recs NOT Met (*n* = 115)		Sleep Recs Met (*n* = 130)			
		Mean	SD	Mean	SD	Mean	SD	OR (95%CI) *	
Three Factor Eating Questionnaire									
Cognitive restraint (0 to 100) *n* = 227	0.81	40.75	22.40	41.28	23.36	40.28	21.60	1.00 (0.99, 1.01)	0.936
Emotional eating (0 to 100) *n* = 238	0.86	38.82	31.04	45.15	33.50	33.42	27.79	0.99 (0.98, 1.00)	0.062
Uncontrolled eating (0 to 100) *n* = 232	0.87	40.50	21.61	43.52	23.51	37.87	19.53	0.99 (0.98, 1.01)	0.156
Total Fruit and Vegetables Servings/day (*n* = 219)	n/a	2.44	1.82	2.44	1.82	2.45	1.95	1.02 (0.88, 1.18)	0.808
Total Caffeine Intake ^A^ (servings/week)	n/a	2.96	2.62	3.19	2.62	2.75	2.61	0.95 (0.86, 1.05)	0.346
Total Alcohol Intake ^B^ (servings/week) (*n* = 241)	n/a	1.48	2.45	1.33	2.22	1.62	2.63	1.06 (0.95, 1.19)	0.278
Physical Activity Level ^C^ (0 to 49)	n/a	15.85	11.03	15.21	11.12	16.42	10.96	1.01 (0.99, 1.04)	0.328

Note: Recs = Recommendations, * Adjusted odds ratio and 95% confidence intervals were calculated using binary logistic regression analysis for each outcome controlling for BMI; ^A^ total caffeine intake was sum of coffee, espresso, tea, soft drinks, and energy drinks servings per week (possible score range 0 to 25); ^B^ total alcohol intake was the sum of beer, wine, liquor, and mixed drinks per week (possible score range 0 to 20); ^C^ physical activity index score derived from the International Physical Activity Questionnaire = (# days of vigorous activities per week × 3) + (# days of moderate activities × 2) + # days of walking 10 min at a time + # days of strength training (possible score range = 0 to 49).
